# Bacterial Community Diversity Associated With Different Utilization Efficiencies of Nitrogen in the Gastrointestinal Tract of Goats

**DOI:** 10.3389/fmicb.2019.00239

**Published:** 2019-02-20

**Authors:** Lizhi Wang, Kaizhen Liu, Zhisheng Wang, Xue Bai, Quanhui Peng, Lei Jin

**Affiliations:** Institute of Animal Nutrition, Sichuan Agricultural University, Chengdu, China

**Keywords:** goat, utilization efficiency of nitrogen, high-throughput sequencing, gastrointestinal tract, bacteria

## Abstract

The objective of this study was to examine the association between bacterial community structure and the utilization efficiency of nitrogen (UEN) phenotypes by determining the bacterial community in the gastrointestinal tract (GIT) of goats that differ in UEN using high-throughput 16S rRNA gene sequencing. Thirty Nubian goats were selected as experimental animals, and their UEN was determined in a metabolic experiment. Subsequently, eight individuals were grouped into the high nitrogen utilization (HNU) phenotype, and seven were grouped into the low nitrogen utilization (LNU) phenotype. The bacterial 16S rRNA gene amplicons from the rumen, abomasum, jejunum, cecum and colon contents of these animals were sequenced using next-generation high-throughput sequencing technology. Two hundred thirty-nine genera belonging to 23 phyla in the rumen, 319 genera belonging to 30 phyla in the abomasum, 248 genera belonging to 36 phyla in the jejunum, 248 genera belonging to 25 phyla in the colon and 246 genera belonging to 23 phyla in the cecum were detected, with Bacteroidetes and Firmicutes predominating. In addition, a significant correlation was observed between the UEN and the genera *Succiniclasticum, Bacteroides, Ruminobacter, Methanimicrococcus, Mogibacterium, Eubacterium_hallii_group* and *Ruminococcus_1* in the rumen; *Bacteroidales_S24-7_group, Bacteroidales_RF16_group, Bacteroidales_UCG-001* and *Anaerovibrio* in the abomasum; *Ruminococcus_2, Candidatus_Saccharimonas, Candidatus_Arthromitus* and *Coprococcus_1* in the jejunum; *Erysipelotrichaceae_UCG-004, Akkermansia, Senegalimassilia, Candidatus_Soleaferrea* and *Methanocorpusculum* in the colon; and *Ruminococcaceae_UCG-002, Anaerovibrio and Ruminococcaceae_UCG-007* in the cecum. Furthermore, the real-time PCR results showed that the ruminal copies of *Fibrobacter_succinogenes*, *Butyrivibrio_fibrisolvens*, *Ruminococcus*_sp._HUN007, *Prevotella ruminicola* and *Streptococcus bovis* in the HNU animals were significantly higher than those in the LNU animals. This study suggests an association of GIT microbial communities as a factor that influences UEN in goats.

## Introduction

The rapid development of animal husbandry has not only improved the living conditions of humans but also led to serious environmental problems caused partially by the excessive discharge of nitrogen in feces and urine from livestock. Statistics have shown that animal production discharges 80–130 Tg (million tons) of nitrogen annually worldwide, which is equivalent to the amount of global nitrogen fertilizer use (National Register of Citizens [NRC], 2003). The excreted nitrogen can be oxidized into nitrate and infiltrated into soil and water, resulting in the acidification of soil, eutrophication of water and loss of biodiversity. Therefore, it is of great significance to solve the environmental nitrogen pollution caused by animal production.

The serious nitrogen pollution caused by animal production can be primarily attributed to low nitrogen utilization (LNU). Most ingested dietary nitrogen is not effectively utilized by the host but excreted into the environment. Compared with pigs and fowl, the utilization efficiency of dietary nitrogen is lower for ruminants and less than 25% in dairy cows, which are considered to be the ruminants with the highest efficiency of nitrogen (UEN) (Kohn et al., 2005). Statistical analyses indicated that 60% of the nitrogen discharged from global animal production is derived from cattle and 12% is derived from sheep (Oenema and Tamminga, 2005).

In recent decades, to improve dietary UEN in ruminants, researchers have proposed and established a new system of protein nutrition in ruminants based on rumen-degradable protein (RDP) and rumen-undegradable protein (RUP), and various nutrient regulation methods have been developed, such as balancing the proportions of RDP and RUP and feeding rumen-bypass amino acids. Statistics have shown that the average nitrogen utilization of American cows was 23.70% in 1958 (Stone et al., 1960) and only 24% 48 years later (Huhtanen and Hristov, 2009) despite the wide use of the nutrition balance technique based on the new protein system in dietary formulations. This phenomenon indicated that the existing nutritional technology used to improve UEN is ineffective. Moreover, a wide range of variation (15–40%) in UEN was noticed among experiments (Calsamiglia et al., 2010; Hester and Harrison, 2012). The variation reflected differences in feeding practices or experimental conditions, suggesting that improvements are possible through the modification of factors regulating key processes of the UEN. These factors are multifarious, and some of them may not yet be understood, such as the impact of microbes in the gastrointestinal tract (GIT). Many previous studies have shown that the bacterial communities across the GIT of mammals provide important benefits to their hosts (Savage, 1977; Ley et al., 2008; Sommer and Bäckhed, 2013), including improvements in the digestion and utilization of host nutrients (Ridaura et al., 2013; Mao et al., 2015), and the bacteria in the rumen of ruminants are especially noted (Wang et al., 2017). In the present study, we hypothesized that the gastrointestinal microbial community could significantly affect the dietary UEN of the host. In order to assess the validity of such hypothesis and evaluate the association of the microbial community profile with the variation of UEN in goats, this study aimed at characterizing the GIT bacterial community of goats differing in UEN.

## Materials and Methods

The animal experimental procedures were approved by the Animal Policy and Welfare Committee of the Agricultural Research Organization of Sichuan Province, China and conducted in accordance with the guidelines of the Animal Care and Ethical Committee of Sichuan Agricultural University.

### Animals and Sampling

This trial was performed at the Animal Nutrition Institute, Sichuan Agricultural University. Thirty healthy female Nubian goats of the same age were chosen as the experimental animals from a flock of goats raised under the same environmental conditions at Pangzhihua He Xie farm, Sichuan Province, China. Each of the 30 goats was housed in separated pens during the 30-day prefeeding and 6-day metabolism experiment. All goats were fed the same total mixed ration containing 55% forage and 45% concentration (Table 1) with free access to water. A restricted diet (4% body weight, DM) was provided daily. All feces and urine were collected during the metabolism experiment, and 10% of the samples were sampled randomly every day and then mixed with 10% volume 10% HCL for nitrogen fixation. The daily feed intake and residual intake were recorded within 6 days for the subsequent calculation of the nutritional composition and UEN.

**Table 1:** Composition and nutritional level of the diet.

Ingredients	Content (%, DM basis)
Corn	38.47
Soybean meal	4.50
Saleratus	0.45
NaCl	0.45
Ca_2_CO_3_	0.08
CaHPO_4_	0.60
Premix^1^	0.45
Alfalfa meal	20.00
Guinea grass	35.00
Total	100.00
Nutrition levels^2^	
Metabolizable energy (MJ/kg DM)	9.34
Crude Protein	9.71
Calcium	0.52
Phosphorus	0.33

*^1^Premix provides the following nutrients per kg of diet: Fe (as ferrous sulfate), 40 mg; Cu (as copper sulfate), 10 mg; Zn (as zinc sulfate), 70 mg; Mn (as manganese sulfate), 40 mg; VA, 2937 IU; VD, 343 IU; and VE, 30 IU. ^2^Nutrition levels are measured values except for ME, which is a calculated value.*

All goats were slaughtered before morning feeding on the second day after the metabolism experiment, and the rumen, abomasum, jejunum, cecum and colon were obtained and separated to prevent shifting of luminal contents during transport from one site to another. Eight tubes (50 mL each) of rumen contents and four tubes (15 mL each) of abomasum, jejunum, cecum and colon digesta were collected. Ruminal aliquots were collected to evaluate the pH as well as the concentrations of rumen ammonia N and volatile fatty acids (VFAs; acetate, propionate, and butyrate). These samples were filtered through a triple cheesecloth layer and subjected to pH evaluation (PHS-100 portable acidity meter, Tianqi Mdt InfoTech Ltd., Shanghai, China). Next, a 20-mL rumen fluid was fixed with 1 mL of H_2_SO_4_ (1:1) and frozen (-20°C) for an ammonia N concentration analysis. A second 20-mL aliquot was fixed with 5 mL of meta-phosphoric acid solution (250 g/L) and kept at -20°C for assessment of the VFA concentration. The remaining rumen and other gastrointestinal section samples were kept at -80°C for subsequent measurements.

### Analysis of Samples and Grouping

All samples of feed and feces were dried in a forced-air oven at 65°C for 48 h to measure the dry matter (DM) and then ground to pass through a 40-mesh sieve. The crude protein (CP) contents were determined by the Kjeldahl method, the ether extract (EE) content was determined by the Soxhlet extraction method, and the organic matter (OM) and crude ash contents were measured in a muffle furnace at 550°C for 6 h (Association of Official Analytical Chemist [AOAC], 1990). The neutral detergent fiber (NDF) and acid detergent fiber (ADF) contents were determined as described by Van Soest et al. (1991) using the filter bag technique without sodium sulfite and expressed with residual ash. The samples of urine were analyzed for CP as described for the feed samples. The rumen fluid was centrifuged (12,000 ×*g* for 10 min at 4°C), and the supernatant was harvested for the detection of VFAs, ammonia N (N-NH_3_) and microorganism crude protein (MCP). The concentrations of VFAs (acetate, propionate and butyrate) were measured using gas chromatography (GC-2014FRGA1, Shimadzu, Tokyo, Japan) (Cottyn and Boucque, 1968), and the MCP and N-NH_3_ concentrations were quantified using a colorimetric technique (Bhandari et al., 2007). The UEN was calculated as follows:

UEN(%)=(A*B−C*D−E*F)/A*B*100.

where A is the feed intake, which is calculated as the amount of supplied feed minus the residual intake during the 6-day metabolism experimental period; B is the CP concentration of the feed; C is the total amount of feces during the 6-day metabolism experimental period; D is the CP concentration of feces; E is the total amount of urine in the 6-day metabolism experimental period; and F is the CP concentration in urine.

The mean and standard deviation (SD) of the UEN of the 30 goats were calculated. Then, the SD values above and below the mean were used to group animals into the high nitrogen utilization (HNU, UEN > mean + 0.5 ^∗^ SD) phenotype and low nitrogen utilization (LNU, UEN < mean – 0.5 ^∗^ SD) phenotype based on a previously described method (Nkrumah et al., 2006; Ramos and Kerley, 2013).

The digestibility of DM, ASH, OM, EE, NDF, and ADF were calculated as follows (Niu et al., 2015):

X digestibility(%) = (A*B−C*D)/A*B*100.

where X = DM, ASH, OM, EE, NDF or ADF,

A is the feed intake, which is calculated as the amount of supplied feed minus the residual intake during the 6-day metabolism experimental period; B is the X concentration in the feed; C is the total amount of feces during the 6-day metabolism experimental period; and D is the X concentration in feces.

### DNA Extraction

Before DNA extraction, the ruminal contents were transferred to the four layers of gauze and strained through it. The fluid was collected into another sterile EP tube and immediately centrifuged at 10,000 ×*g*. The supernatants were removed and the remaining samples were used for DNA extraction.

In addition to the ruminal samples, the other GIT homogenized digesta acquired from the two groups were also used for DNA extraction using the TIANamp Bacteria DNA Kit (TIANGEN, Peking, China) according to the manufacturer’s guidelines as described previously (Guo et al., 2015). Then, the quality of the DNA samples was determined by agarose electrophoresis and a Nanodrop 8000 spectrophotometer (Thermo Fisher Scientific, Brisbane, QLD, Australia).

### PCR Amplification and Sequencing

The 16S rDNA V4 hypervariable regions of the total microbial genomic DNA were used for PCR amplification with the universal primers 515F (5′-GTGCCAGCMGCCGCGGTAA-3′) and 806R (5′-GGACTACHVGGGTWTCTAAT-3′) (Meale et al., 2017). All PCR amplifications were performed in triplicate in 30-μL reactions (PCR thermal cycler Model C1000, Bio-Rad, Richmond, CA, United States) that consisted of 3 μL of each primer (2 μM), 15 μL of Phusion Master Mix, 10 μL of 1 ng/μL template DNA and Milli-Q water to a final volume of 30 μL. The amplification was initiated with a denaturation at 94°C for 3 min; 30 cycles of denaturation at 94°C for 30 s, 50°C for 30 s, and 72°C for 30 s; and a final extension at 72°C for 5 min. Finally, the three replicates of DNA extracted from each sample were mixed together.

The products were purified using a PCR Clean-Up system (Promega, Madison, WI, United States) with a purification kit (QIAGEN, Adelaide, SA, Australia) and quantified using a QuantiFlour^TM^-ST fluorometer (Promega, Madison, WI, United States). Subsequently, the amplicons of each reaction mixture were pooled into a single tube in equimolar ratios to generate the amplicon libraries. Before the samples were pooled at equal volumes, each amplicon library was first diluted to 1^∗^10^9^ molecules/mL. Then, the pooled amplicon libraries were diluted to 1^∗^10^7^ molecules/mL. Finally, the samples were sequenced on an Illumina MiSeq HiSseq 2500 Sequencing Platform.

### Sequencing Data Processing

QIIME pipeline software (version 1.8.0) was used to analyze the reads acquired from Macrogen Inc. (Caporaso et al., 2010b). Low-quality sequences, such as sequences containing uncertain nucleotides, three continuous nucleotides with Q values less than 20 and unmatched barcode sequences, were removed. Chimeric sequences were removed using Usearch V7.0 based on the Uchime algorithm implemented in QIIME (Edgar, 2010). Clean and high-quality sequences were then clustered into operational taxonomic units (OTUs) based on 97% similarity. The most abundant sequence was selected as the representative for each OTU and then aligned against the Greengenes database^1^ using PyNAST (Caporaso et al., 2010a; Koetschan et al., 2014). Taxonomic OTU assignments were performed using the RDP Classifier.

Alpha diversity indices (Shannon-Wiener, PD_whole_tree, Chao1 and Observed-species) were calculated at a depth of 21,854 sequences. Beta diversity was visualized using a principal coordinate analysis (PCoA) as measured using an unweighted UniFrac distance matrix (Jin et al., 2017). In addition, genera that were shared by all the samples were selected to create a heat map using the software program R version 3.0.2 (Lozupone et al., 2011; Jami et al., 2013). All sequence data in the present study were deposited in the Sequence Read Archive (SRA) of the NCBI database under number PRJNA290544.

### Real-Time PCR Analysis

Copy numbers of seven special ruminal bacteria associated with protein degradation were quantified using real-time PCR, which was performed in a Bio-Rad cfx96 thermo cycler (Bio-Rad, Hercules, CA, United States). The primers are shown in Table 2. The real-time PCR utilized a SYBR Premix Ex Taq II (Tli RNaseH Plus) assay kit (TaKaRa Bio Inc., Shiga, Japan) and was implemented with the standard curve method. The standard curves were generated by diluting plasmid DNA (10-fold serial dilution) containing the cloned marker loci (Stubner, 2002). The assays included DNA samples from 15 goats in the two groups. Three technical replicates of each sample were implemented.

**Table 2:** Primers used for the RT-PCR assays.

Bacteria	Primers (5′–3′)	Reference
*Ruminococcus*_sp._HUN007	AGGCGGGACTGTAAGTCAGA	Self-designed
	ACGCATTTCACCGCTACACT	
*Fibrobacter_succinogenes*	GGCGGGATTGAATGTACCTTGAGA	Self-designed
	TCCGCCTGCCCCTGAACTATC	
*Butyrivibrio_fibrisolvens*	TAACATGAGAGTTTGATCCTGGCTC	Dan, 2008
	CGTTACTCACCCCCCCCGTCCGC	
*Prevotella_ruminicola*	GAAAGTCGGATTATGCTCTATGTTG	Wang et al., 2017
	CATCCTATAGCGGTAAACCTTTGG	
*Succinivibrio_dextrinosolvens*	TAGGAGCTTGTCGATAGTATGG	Wang et al., 2017
	CTCACTATGTCAAGGTCAGGTAAGG	
*Ruminococcus_flavefaciens*	CGAACGGAGATAATTTGAGTTTACTTAGG	Wang et al., 2017
	CGGTCTCTGTATGTTATGAGGTATTACC	
*Streptococcus bovis*	TTCCTAGAGATAGGAAGTTTCTTCGG	Li et al., 2014
	ATGATGGCAACTAACAATAGGGGT	

The 25-μL reaction mixture consisted of 1 μL (each) of forward and reverse primers, 12.5 μL of 2× SYBR Premix Ex Taq II (Tli RNaseH Plus) (TaKaRa Bio Inc., Shiga, Japan), and 5 μL of rumen DNA sample. The following thermal cycling program was employed: 10 min for an initial denaturation step at 95°C; 40 cycles of denaturation at 95°C for 15 s, annealing for 30 s, and elongation at 72°C for 45 s; and a final extension at 72°C for 10 min. All standard curves met the following requirements [the coefficient of determination (*R*^2^) values of all the standard curves were greater than 0.99].

### Statistical Analysis

The non-parametric test of two samples was performed using SPSS Statistics software v. 19.0 (IBM, Armonk, NY, United States) to assess differences in UEN, bacterial relative abundance and other parameters between the HNU and LNU groups. The results are shown as the mean ± SD. A Spearman rank correlation analysis between the relative abundance of bacteria and UEN was performed using SPSS Statistics software v.19.0 (IBM, Armonk, NY, United States). Significant and extremely significant levels were set at *P* < 0.05 and *P* < 0.01, respectively.

## Results

### UEN of Goats

The UEN of the thirty goats exhibited a high variation (from 20.71 to 67.47%), with an average of 45.33% ± 13.54% (Supplementary Table S1). In this trial, eight individuals were grouped as the HNU phenotype and seven as the LNU phenotype.

The difference in UEN and the apparent digestibility of CP between the HNU and LNU animals were significant (*P* < 0.05) (Supplementary Tables S1, S2). However, significant differences were not observed in the apparent digestibility of DM, OM, ASH, EE, NDF, and ADF between the two groups (*P* ≥ 0.05) (Supplementary Table S2).

### Index of Ruminal Fermentation

**FIGURE 1:**
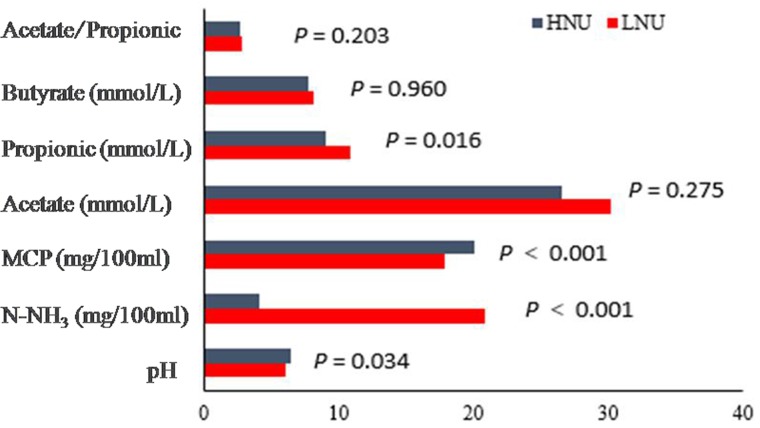
Comparison of the ruminal fermentation indices between the HNU and LNU groups.

The indices of ruminal fermentation are presented in Figure 1. The pH value (*P* = 0.034) and the concentrations of MCP (*P* < 0.001) were significantly higher in the HNU goats than the LNU goats, whereas the concentrations of N-NH_3_ (*P* < 0.001) and propionate (*P* = 0.016) were significantly lower in the HNU than LNU animals. The concentrations of acetate (*P* = 0.275), butyrate (*P* = 0.960) and acetate/propionic (*P* = 0.203) between the two groups showed no significant differences.

### Data Acquired From Sequencing and Alpha-Diversity Measures

**FIGURE 2:**
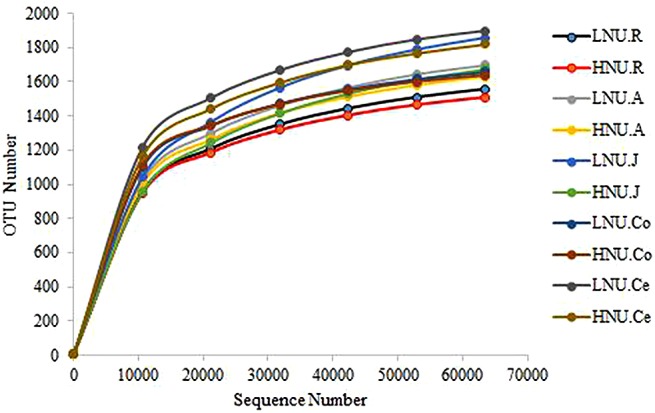
Rarefaction curves of the groups. HNU-R, rumen of HNU; LNU-R, rumen of LNU; HNU-A, abomasum of HNU; LNU-A, abomasum of LNU; HNU-J, jejunum of HNU; LNU-J, jejunum of LNU; HNU-Co, colon of HNU; LNU-Co, colon of LNU; HNU-Ce, cecum of HNU; LNU-Ce, cecum of LNU. The same below.

In the sequencing analysis of 16S rRNA, a total of 5,711,204 high-quality sequences were generated, with an average of 78,679 ± 5,548 per sample in the rumen, 76,694 ± 5,082 in the abomasum, 74,780 ± 6,257 in the jejunum, 74,799 ± 5,715 in the colon, and 75,793 ± 4,588 in the cecum. At a 97% nucleotide sequence identity between reads, the overall number of OTUs detected by the analysis was 140,403, with an average of 1,690 ± 158 per sample in the rumen, 1,851 ± 212 in the abomasum, 1,987 ± 343 in the jejunum, 1,787 ± 211 in the colon, and 2,044 ± 249 in the cecum (Supplementary Figure S1). The rarefaction curve (Figure 2) and the value of Good’s coverage (Table 3) indicated that the sampling had sufficient sequence coverage to detect the majority of microorganisms. The richness and evenness of the microbiota across GIT as indicated by the alpha diversity indices (PD_whole_tree, Chao1, Shannon, Observed_species, Simpson and ACE) were not significantly different (*P* > 0.05; Table 3) between groups.

**Table 3:** Comparison of the alpha diversity indices between the HNU and LNU groups.

Indices	Region	LNU (*n* = 7)	HNU (*n* = 8)	*P*-value
Observed_Species	Rumen	1557.42 ± 187.62	1507.75 ± 77.00	0.503
	Abomasum	1697.43 ± 273.59	1632.25 ± 104.02	0.542
	Jejunum	1855.43 ± 344.73	1675.38 ± 276.24	0.282
	Colon	1660.29 ± 247.86	1636.63 ± 134.38	0.818
	Cecum	1895.86 ± 179.90	1819.63 ± 299.07	0.568
Shannon	Rumen	7.60 ± 0.38	7.69 ± 0.37	0.646
	Abomasum	7.57 ± 0.71	7.84 ± 0.20	0.333
	Jejunum	7.11 ± 1.10	6.94 ± 1.20	0.786
	Colon	8.24 ± 0.49	8.20 ± 0.79	0.908
	Cecum	8.45 ± 0.47	7.97 ± 1.99	0.546
Simpson	Rumen	0.98 ± 0.02	0.98 ± 0.01	0.813
	Abomasum	0.97 ± 0.03	0.98 ± 0.005	0.132
	Jejunum	0.96 ± 0.04	0.95 ± 0.04	0.816
	Colon	0.99 ± 0.01	0.97 ± 0.04	0.525
	Cecum	0.99 ± 0.01	0.93 ± 0.08	0.398
Chao1	Rumen	1708.94 ± 214.86	1641.50 ± 93.26	0.434
	Abomasum	1848.78 ± 287.18	1780.22 ± 123.29	0.548
	Jejunum	2066.86 ± 367.78	1870.22 ± 311.49	0.282
	Colon	1780.95 ± 290.00	1739.51 ± 142.64	0.725
	Cecum	2043.43 ± 199.22	1969.33 ± 311.73	0.599
ACE	Rumen	1712.56 ± 217.18	1648.18 ± 87.19	0.453
	Abomasum	1879.35 ± 291.50	1796.49 ± 129.84	0.479
	Jejunum	2100.04 ± 372.41	1892.87 ± 310.70	0.261
	Colon	1785.37 ± 293.88	1744.06 ± 143.57	0.729
	Cecum	2058.41 ± 196.47	1982.2 ± 308.23	0.585
Goods_Coverage	Rumen	0.99628 ± 0.00075	0.99650 ± 0.00053	0.533
	Abomasum	0.99557 ± 0.00053	0.99587 ± 0.00064	0.342
	Jejunum	0.99443 ± 0.00098	0.99513 ± 0.00146	0.304
	Colon	0.996571 ± 0.00127	0.99688 ± 0.00035	0.521
	Cecum	0.99571 ± 0.00076	0.99588 ± 0.00064	0.663
PD_Whole_Tree	Rumen	96.48 ± 7.84	94.87 ± 4.10	0.621
	Abomasum	104.68 ± 10.60	102.79 ± 5.07	0.659
	Jejunum	114.26 ± 17.75	104.52 ± 12.95	0.242
	Colon	89.02 ± 14.97	90.39 ± 7.12	0.820
	Cecum	101.88 ± 8.00	99.84 ± 12.38	0.716

### OTU Diversity and Similarity Analysis

**FIGURE 3:**
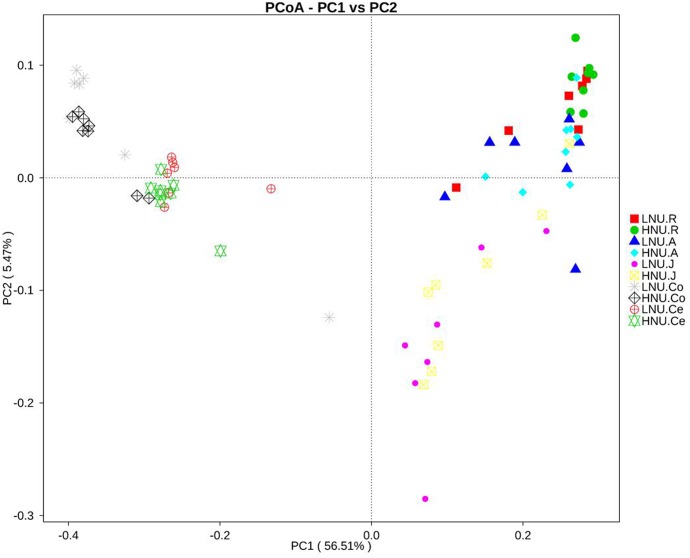
Principal coordinate analysis of bacterial samples from the gastrointestinal tract.

Comparisons of the OTUs between the HNU and LNU groups across the GIT were visualized using a PCoA plot with unweighted UniFrac distance metrics (Figure 3). A closer distance between two points indicated a greater similarity of the samples, and the percentage of variation was elucidated by PC1 and PC2 as indicated by the axis. In the present study, PC1 explained 56.51% of the variation and PC2 explained 5.47%. The PCoA plot showed no obvious boundary between the microbial communities of the HNU and LNU groups. The samples from the same/adjacent GIT region (rumen, jejunum and large intestine) were more similar to each other than to those from other regions.

### Bacterial Composition of the GIT

**FIGURE 4:**
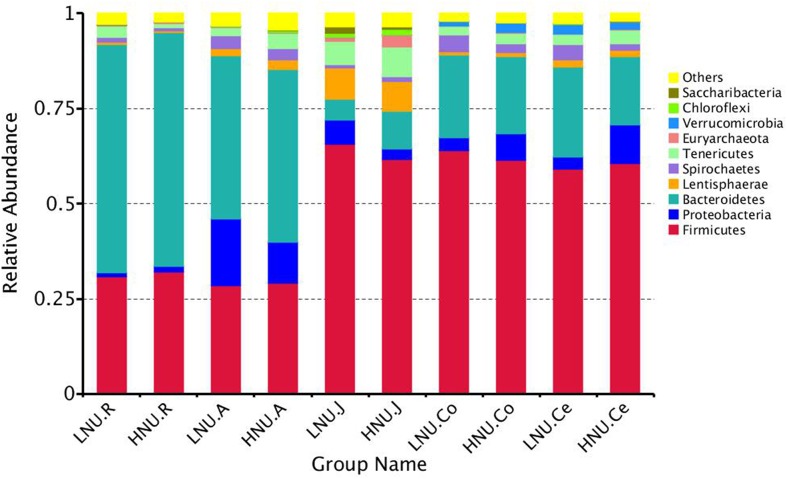
Bacterial compositions across the gastrointestinal tracts at the phylum level. (Only the top 10 abundant phyla are presented).

At the phylum level, 23 taxa were identified in the rumen and cecum, 30 in the abomasum, 36 in the jejunum and 25 in the colon. Figure 4 shows the top 10 abundant phyla in all groups. The most abundant phylum in the rumen was Bacteroidetes (HNU 61.37%, LNU 59.91%), and the secondary phylum was Firmicutes (HNU, 32.11%; LNU, 30.75%). In the abomasum, the majority of the obtained sequences belonged to Bacteroidetes (HNU, 45.59%; LNU, 42.89%), Firmicutes (HNU, 29.16%; LNU, 28.42%) and Proteobacteria (HNU, 10.66%; LNU, 17.66%). Firmicutes (HNU, 61.70%; LNU, 65.57%) was the dominant phylum in the jejunum, followed by Lentisphaerae (HNU, 7.91%; LNU, 8.16%), Bacteroidetes (HNU, 9.86%; LNU, 5.58%) and Tenericutes (HNU, 7.98%; LNU, 6.10%). The dominant bacteria in the colon and cecum were similar, and the most abundant phyla were Firmicutes (average of 63.76% in colon, 59.83% in cecum), Bacteroidetes (average of 20.99% in colon, 50.58% in cecum) and Proteobacteria (average of 5.11% in colon, 6.73% in cecum) in both sites.

**FIGURE 5:**
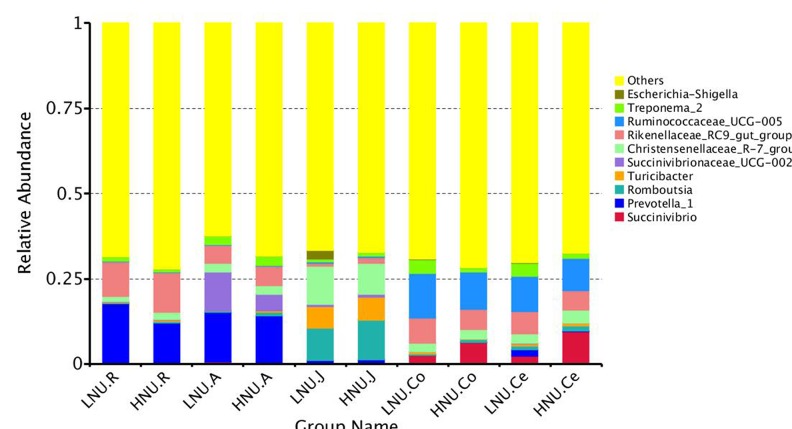
Bacterial compositions across the gastrointestinal tracts at the genus level. (Only the top 10 abundant genera are presented).

**FIGURE 6:**
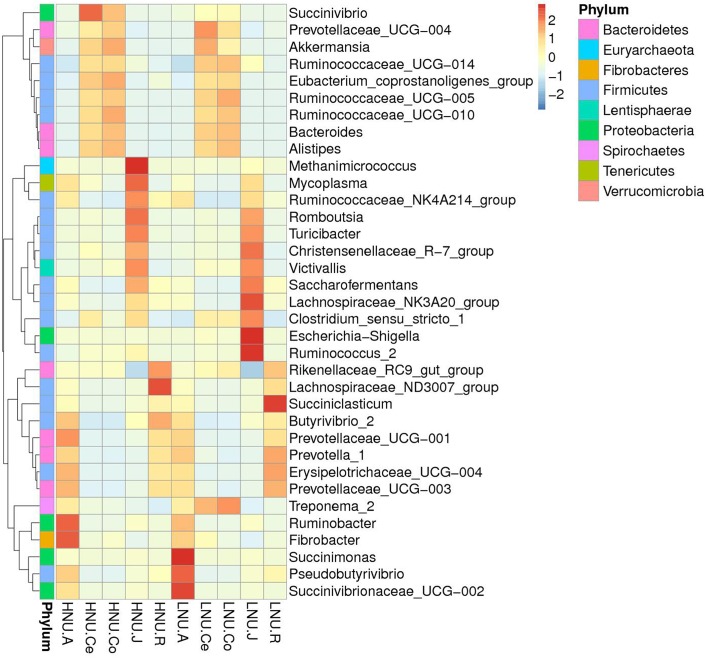
Heatmap of the core genera across the gastrointestinal tracts (the relative abundance of microbes was log-transformed).

A total of 239 genera were detected in the rumen, 319 in the abomasum, 248 in the jejunum, 248 in the colon, and 246 in the cecum. Among the obtained sequences, an average of 38.19% in the rumen, 29.93% in the abomasum, 23.45% in the jejunum, 27.25% in the colon, and 26.53% in the cecum were not identified at the level of genus. The average relative abundances of the top 10 abundant genera are shown in Figure 5. The dominant genera in the rumen were *Prevotella_1* (HNU, 12.02%; LNU, 17.63%) and *Rikenellaceae_RC9_gut_group* (HNU, 11.71%; LNU, 10.16%). In the abomasum, *Prevotella_1* (HNU, 13.94%; LNU, 14.61%), *Succinivibrionaceae_UCG-002* (HNU, 4.92%; LNU, 11.42%) and *Rikenellaceae_RC9_gut_group* (HNU, 5.70%; LNU, 5.266%) were the three most abundant genera, similar to the rumen. Compared with the rumen and abomasum, the dominant genera in the jejunum, colon and cecum were very different. Since *Romboutsia* (HNU, 11.54%; LNU, 9.56%), *Christensenellaceae_R-7_group* (HNU, 9.11%; LNU, 11.15%) and *Turicibacter* (HNU, 6.65%; LNU, 6.15%) were the most abundant genera in the jejunum, *Ruminococcaceae_UCG-010* (in colon: HNU, 11.18% and LNU, 9.73%; in cecum: HNU, 8.62% and LNU, 8.36%) and *Ruminococcaceae_UCG-005* (in colon: HNU, 10.978% and LNU, 12.96%; in cecum: HNU, 9.59% and LNU, 10.35%) were the two primary genera in the colon and cecum. The shared genera across the GIT were chosen and are shown in the heatmap (Figure 6). Within the abovementioned genera, *Prevotella_1* and *Rikenellaceae_RC9_gut_group* belong to the phylum Bacteroidetes, *Succinivibrionaceae_UCG-002* is a member of Proteobacteria, and the remaining genera are from the phylum Firmicutes.

### Comparisons of Bacterial Composition Across the GIT Between the HNU and LNU Groups

When the relative abundance of bacteria from the phylum to genus level was compared, a number of bacteria were detected to have significant differences between the HNU and LNU groups (Table 4), and the numbers of these bacteria in the rumen were greater than that in the jejunum, abomasum, colon and cecum. In the rumen, the relative abundance of the dominant bacterium *Prevotella* and *Succiniclasticum* was significantly higher (*P* < 0.05) in the LNU than the HNU animals. The relative abundance of *Ruminococcus_1*, *Lachnospiraceae_XPB1014_group*, *Ruminococcaceae_UCG-004* and *Ruminobacter* was significantly higher (*P* < 0.05) in the HNU than the LNU group. In the abomasum, *Eubacterium_nodatum_group*, *Anaerovibrio* and *Methanobrevibacter* had significantly different relative abundances between the two groups; however, their relative abundance was fairly low. In the jejunum, the relative abundance of *Ruminococcus_2* was significantly higher (*P* < 0.05) in the LNU than the HNU. In the colon, *Anaeroplasma* and *Lachnospiraceae*_UCG-009 were significantly higher (*P* < 0.05) in the LNU group, although *Blautia* and *Parvibacter* were significantly lower (*P* < 0.05). In the cecum, the relative abundance of *Prevotellaceae_UCG-004* and *Thalassospira* was higher (*P* < 0.05) in the LNU than HNU animals, although unidentified_Ruminococcaceae and *Allobaculum* were significantly lower (*P* < 0.05).

**Table 4:** Microbial taxa with relative abundances that were significantly different between HNU and LNU across the GITs.

			Relative abundance (%)		
Region	Level	Taxa	LNU (*n* = 7)	HNU (*n* = 8)	*P-*value
Rumen	Class	Clostridia	20.360 ± 2.272	26.504 ± 4.866	0.009
		Negativicutes	7.597 ± 4.376	3.309 ± 1.966	0.026
		Betaproteobacteria	0.061 ± 0.015	0.088 ± 0.025	0.023
		Unidentified_Firmicutes	0.008 ± 0.003	0.014 ± 0.004	0.004
	Order	Clostridiales	20.312 ± 2.267	26.425 ± 4.844	0.009
		Selenomonadales	7.597 ± 4.376	3.309 ± 1.966	0.026
		Anaeroplasmatales	0.052 ± 0.010	0.071 ± 0.017	0.020
	Family	Bacteroidales_S24-7_group	7.888 ± 2.645	16.900 ± 5.678	0.002
		Ruminococcaceae	7.817 ± 0.567	9.294 ± 1.004	0.004
		Acidaminococcaceae	5.611 ± 3.767	1.702 ± 1.008	0.014
		Anaeroplasmataceae	0.052 ± 0.010	0.071 ± 0.017	0.020
		Family_XIII	0.007 ± 0.003	0.013 ± 0.005	0.006
	Genus	*Prevotella*	17.63 ± 5.036	12.023 ± 4.751	0.045
		*Succiniclasticum*	5.570 ± 3.670	1.702 ± 1.008	0.013
		*Ruminococcus*	0.862 ± 0.248	1.257 ± 0.311	0.018
		p-1088-a5_gut_group	0.614 ± 0.426	0.244 ± 0.188	0.044
		Lachnospiraceae_XPB1014_group	0.244 ± 0.066	0.408 ± 0.140	0.014
		Ruminococcaceae_UCG-004	0.137 ± 0.046	0.224 ± 0.054	0.005
		*Ruminobacter*	0.074 ± 0.075	0.222 ± 0.134	0.024
		Family_XIII_UCG-002	0.064 ± 0.029	0.111 ± 0.042	0.029
		*Anaeroplasma*	0.052 ± 0.010	0.071 ± 0.017	0.020
		Possible_genus_Sk018	0.015 ± 0.008	0.027 ± 0.012	0.045
		*Hydrogenoanaerobacterium*	0.011 ± 0.008	0.004 ± 0.002	0.033
		Lachnospiraceae_FE2018_group	0.002 ± 0.002	0.007 ± 0.005	0.037
		Blvii28_wastewater-sludge_group	0.002 ± 0.002	0.006 ± 0.004	0.045
		*Schwartzia*	0.007 ± 0.004	0.002 ± 0.004	0.022
Abomasum	Genus	*Eubacterium_nodatum_group*	0.050 ± 0.012	0.069 ± 0.017	0.030
		*Anaerovibrio*	0.057 ± 0.023	0.034 ± 0.010	0.026
		*Methanobrevibacter*	0.022 ± 0.012	0.041 ± 0.019	0.044
Jejunum	Class	Epsilonproteobacteria	0.004 ± 0.004	0.001 ± 0.001	0.026
	Order	Neisseriales	0.024 ± 0.018	0.010 ± 0.008	0.310
		Campylobacterales	0.004 ± 0.004	0.001 ± 0.001	0.026
	Family	Neisseriaceae	0.024 ± 0.018	0.010 ± 0.008	0.031
	Genus	Ruminococcus_2	5.317 ± 0.4.620	1.602 ± 0.498	0.034
		Tyzzerella_3	0.0191 ± 0.0087	0.008 ± 0.010	0.049
Colon	Phylum	Euryarchaeota	0.019 ± 0.019	0.096 ± 0.091	0.047
	Class	Methanomicrobia	0.004 ± 0.004	0.074 ± 0.085	0.049
	Order	Anaeroplasmatales	0.109 ± 0.031	0.078 ± 0.022	0.042
	Family	Lachnospiraceae	15.966 ± 2.144	12.984 ± 1.543	0.008
		Anaeroplasmataceae	0.109 ± 0.031	0.078 ± 0.022	0.042
	Genus	*Anaeroplasma*	0.109 ± 0.031	0.078 ± 0.022	0.042
		Lachnospiraceae_UCG-009	0.038 ± 0.022	0.014 ± 0.007	0.014
		*Blautia*	0.016 ± 0.004	0.108 ± 0.004	0.024
		*Parvibacter*	0.002 ± 0.002	0.005 ± 0.003	0.035
Cecum	Phylum	Cyanobacteria	0.087 ± 0.603	0.345 ± 0.261	0.042
		Acidobacteria	0.002 ± 0.003	0.006 ± 0.002	0.017
	Class	Melainabacteria	0.867 ± 0.604	0.338 ± 0.260	0.041
		Unidentified_Acidobacteria	0.002 ± 0.003	0.006 ± 0.002	0.037
	Order	Gastranaerophilales	0.869 ± 0.6039	0.338 ± 0.260	0.041
		Rhodospirillales	0.097 ± 0.0408	0.058 ± 0.021	0.034
	Family	Lachnospiraceae	0.070 ± 0.041	0.031 ± 0.015	0.027
	Genus	Prevotellaceae_UCG-004	2.518 ± 0.856	1.465 ± 0.841	0.032
		*Thalassospira*	0.061 ± 0.044	0.030 ± 0.014	0.049
		Unidentified_Ruminococcaceae	0.011 ± 0.008	0.022 ± 0.008	0.029
		*Allobaculum*	0	0.001 ± 0.001	0.044

**FIGURE 7:**
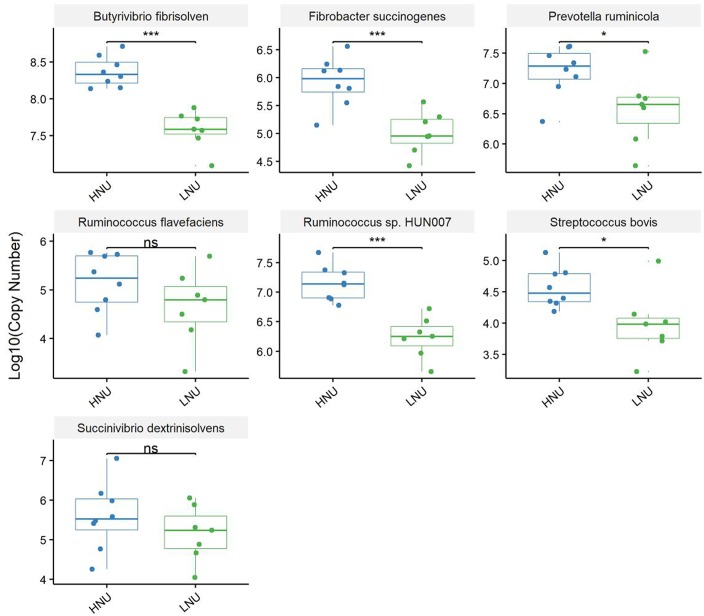
Real-time PCR analysis of ruminal bacteria associated with protein degradation. The *Y*-axis shows the log_10_ of the copies in a l-μl DNA sample. Asterisks indicate an extremely significant difference (^∗∗∗^*P* < 0.001), significant difference (^∗^*P* < 0.05), and “ns” means no significant difference (*P* > 0.05) between the HNU and LNU groups.

Real-time PCR revealed the amounts of *Butyrivibrio_fibrisolvens, Fibrobacter_succinogenes* and *Ruminococcus_*sp*._HUN007* were extremely higher (*P* < 0.001) and *Prevotella ruminicola* and *Streptococcus bovis* were significantly higher (*P* < 0.05) in HNU than LNU. However, *Succinivibrio dextrinisolvens* and *Ruminococcus flavefaciens* showed no significant differences (*P* ≥ 0.05) between the two groups (Figure 7).

### Correlation Between UEN and the Bacterial Composition Across the GIT

**FIGURE 8:**
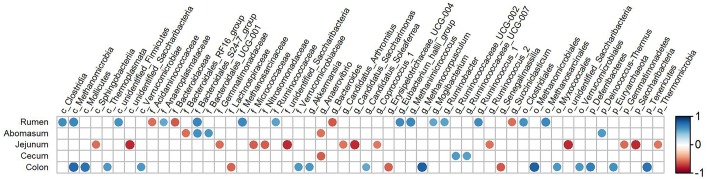
Correlation analysis between the bacteria across the gastrointestinal tracts and the utilization efficiency of nitrogen in goats. Strong correlations are indicated by large circles and weaker correlations by small circles. The scale colors denote whether the correlation is positive (closer to 1, red circles) or negative (closer to –1, blue circles) between the taxa and gastrointestinal tract variables.

To determine the relationship between the UEN and the relative abundance of bacteria in each segment of the GIT, a correlation analysis was performed (Figure 8). The results showed that in the rumen, the relative abundance of three taxa at the class level, two at the order level, six at the family level and seven at the genus level had a tight relationship with the UEN; and in the colon, the relative abundance of three phyla, four classes, three orders, two families and five genera were influenced by the variation of the UEN. Among these microorganisms, class-level Methanomicrobia, order-level Methanomicrobiales, family-level Methanosarcinaceae and genus-level *Methanimicrococcus* belong to the archaea. In the abomasum, only the phylum Deinococcus-Thermus, family Bacteroidales_S24-7_group, Bacteroidales_RF16_group and Bacteroidales_UCG-001 and genus *Anaerovibrio* were affected by the UEN. In the jejunum, the phyla Saccharibacteria, Gemmatimonadetes and Thermomicrobia; classes unidentified_Saccharibacteria and Sphingobacteriia; families unidentified_Saccharibacteria, Nitrosomonadaceae, Gemmatimonadaceae, and Micrococcaceae; genera *Ruminococcus_2*, *Candidatus_Saccharimonas*, *Candidatus_Arthromitus* and *Coprococcus_1* had a significant negative correlation with the UEN.

## Discussion

The N-NH_3_ in the rumen is the final product of protein and non-protein-nitrogen decomposition, and it is also the raw material for the synthesis of MCP (Hall and Huntington, 2008). When the concentration of N-NH_3_ in the rumen exceeds the ability of microorganisms to utilize it, it will be absorbed into the liver through the rumen wall to synthesize urea, which will ultimately be excreted in the urine, resulting in nitrogen waste (Mcdonald, 1954). The concentration of N-NH_3_ in the rumen is mainly affected by the degradation rate of feed nitrogen and the MCP synthesis rate. In this experiment, the feed nitrogen content and nitrogen degradation characteristics of the two groups were the same. However, the results showed that the NH_3_-N concentration in the HNU group was significantly lower than that in the LNU group, which indicated that the MCP synthesis rate in the HNU group was significantly higher than that in the LNU group, thereby reducing the waste of nitrogen in the form of urea. This feature might explain why the rumen MCP concentration and UEN were significantly higher in the HNU group than the LNU group.

The present study showed that the UEN of the thirty Nubian goats varied highly from 20.71 to 67.47%, although their age, genetic background and feeding management were consistent. Although this phenomenon may have been noticed by researchers in previous scientific research studies, prior to our experiment, no formal report on the individual variation in the UEN of livestock had been published. However, previous studies have shown that individual variation in feed efficiency in ruminants are common (Herd and Arthur, 2009; Ramos and Kerley, 2013). The UEN is a vital part of the feed efficiency; therefore, individual variation in the UEN should be a normal phenomenon. Previous studies have shown that the main factors affecting the UEN of animals include the diet and feeding management (Sinclair et al., 2014; Arndt et al., 2015). The present study evaluated, for the first time, the effects of the structure and composition of microorganisms on the host’s UEN.

Nitrogen in feed is mainly decomposed and changed into MCP by microorganisms in the rumen, and then the MCP enters the small intestine and is digested and absorbed by the host (Mcdonald, 1954; Hall and Huntington, 2008). Approximately 70% of the proteins entering the host’s small intestine were MCP. Therefore, the microorganisms in rumen played an important role in the digestibility of feed protein. Compared with the rumen, although the amount of microbes in the abomasum and small intestine is relatively lower, almost all these bacteria can secrete peptidase, and some of them, such as *Bacillus subtilis*, can secrete protease; thus, they can also affect the UEN of the host (Chichlowski et al., 2015). The microorganisms in the large intestine are similar to ruminal to ruminal microorganisms with regard to nitrogen metabolism, and they are able to not only decompose the undigested protein in the chyme of the large intestine and the urea transported to the large intestine through blood recycle into ammonia but also utilize the produced ammonia to resynthesize MCP. However, due to the lack of lysozymes, the MCP synthesized in the large intestine can no longer be utilized by the host and is excreted from the body with feces (Van den Abbeele et al., 2011; Malmuthuge et al., 2014). In summary, all the microbes in different segments of the GIT of ruminants can affect the UEN of their hosts. It is worth noting that the results of this study indicated that rumen microbes might play a more important role in affecting the UEN of the host compared with other parts of the GIT. Indeed, the findings of the present study demonstrated that the microorganisms that were significantly associated with the host UNE had the largest number and a higher relative abundance in the rumen (Figure 4, 8). In this study, *Prevotella* was a dominant genus in the rumen and shared by all samples, which was consistent with previous research (Bekele et al., 2010; Mohammadzadeh et al., 2014). The present study found that the relative abundance of *Prevotella* was significantly lower in the HNU group than the LNU group. *Prevotella* contains a number of bacterial species, such as *Prevotella bryantii, Prevotella brevis* and *Prevotella_ruminicola*, which have their own substrate preferences (Koike et al., 2003). For example, glucose, lactose and cellobiose are mainly fermentative substrates of *Prevotella brevis* (Avgustin et al., 1997), whereas starch and xylan can be used by *Prevotella bryantii* (Purushe et al., 2010). Only *Prevotella_ruminicola* has been considered to have the function of decomposing protein (Wallace et al., 1997). The results of the present study are consistent with previous experiments because real-time quantitative PCR showed that the copies of *Prevotella_ruminicola* were significantly higher in the HNU than the LNU group (Figure 7).

In this study, the relative abundances of the genus-level bacteria *Ruminococcus*_1 and *Ruminococcaceae*_UCG-004 and the species-level bacteria *Ruminococcus_*sp*._HUN00* in the HNU goats were significantly higher in the HNU than the LNU (Table 4 and Figure 7). The absolute value of the copy number of *Ruminococcus flavefaciens* was also higher in the HNU than the LNU group, although the difference was not significant between groups (Figure 7). Previous studies have shown that many microbes in the family Ruminococcaceae are typical fibrous-degrading bacteria, and most of them possess the ability to degrade protein (Smet et al., 1995; Kang et al., 2017). Therefore, enhancing the abundance of these fibrous-degrading bacteria in the rumen could effectively promote the UEN of the host. *Butyrivibrio_fibrisolvens* and *Fibrobacter succinogenes* are also fibrinolytic bacteria with protein-degrading functions, and the real-time PCR results showed that their copy numbers were significant higher in the HNU group than the LNU group. However, not all microorganisms associated with fiber degradation can promote UEN in the host. For example, *Succiniclasticum* can degrade succinic acid produced by rumen fiber decomposition into propionic acid, thus promoting the decomposition of fiber. However, in this study, we found that the relative abundance of *Succiniclasticum* was significantly lower in the HNU group than the LNU group, indicating that the promotion of fiber degradation does not necessarily promote the utilization of nitrogen by the host. A previous study has shown that ruminal *Succinivibrio dextrinisolvens* are also related to the degradation of feed protein (Wang et al., 2017), although their abundance was not significantly different between the HNU and LNU groups in this study.

The jejunum is one of the main sites for the digestion and absorption of all nutrients, including nitrogen. However, chemical digestion is the main form of digestion due to the small number of microbes. Present study demonstrated that correlations between the jejunum bacteria and the host UEN were negative. As a result, over-reproduction of specific microorganisms in the jejunum might not be beneficial to the digestion and utilization of nitrogen by the host. *Ruminococcus*_2 in jejunum was relatively remarkable because of its high relative abundance (HNU = 5.32% ± 4.62%; LNU = 1.60% ± 0.50%). As mentioned above, in the rumen, many microbes in the family Ruminococcaceae such as *Ruminococcus*_1 can promote the digestion and utilization of feed nitrogen by the host. *Ruminococcus*_2 was also one of the unnamed genera in the family Ruminococcaceae, although this study found that its relative abundance in the jejunum was negatively correlated with the host UEN. A previous study showed that the bacteria of Ruminococcaceae obtained nutrients mainly by decomposing fibers and that their fermentation products were mainly glucose and xylose (Zhu et al., 2018). In theory, more vigorous glucose metabolism is more conducive to the uptake and utilization of nitrogen-containing substances by the host. In turn, the higher UEN of the host indicated more abundant Ruminococcaceae. However, this study found that the relative abundance of *Ruminococcus*_2 in the HNU was significantly lower than that in the LNU; so it can be speculated that the biological characteristics of *Ruminococcus*_2 might be different from those of other species in the Ruminococcaceae family.

The caecum and colon are part of the large intestine. In this study, in both the caecum and colon, some microorganisms were found with relative abundances that were significantly different between the HNU and LNU groups (Table 4). Compared with the cecum, colonic microbes might play a more important role in affecting the host UEN because the bacterial taxonomy number, which showed a significant correlation with the host UEN, was 17 in the colon and only three in the cecum (Figure 8). This result might be attributed to the physiological structure of ruminants. Compared with herbivores, such as horse and rabbit, the cecum of goats is less developed. Furthermore, its length is only approximately 1/20 of the colon, and the degree of peristalsis in the cecum is lower than that in the colon; thus, a much smaller amount of chyme reaches the cecum than the colon. Therefore, despite the abundant microorganisms, the digestion and metabolism of nitrogen in the cecum was significantly weaker than that in the colon (Langer and Snipes, 1991). Lachnospiraceae mainly inhabit the GIT of mammals. A previous study has reported that some genera of Lachnospiraceae in the large intestine can produce short-chain fatty acids (especially butyric acid), which are considered to be associated with the prophylaxis of colon cancer (Meehan and Beiko, 2014). Additionally, Lachnospiraceae has also been found to cause diabetes in germ-free mice (Kameyama and Itoh, 2014). The present study showed a higher relative abundance of Lachnospiraceae both in the colon and cecum in the LNU group than the HNU group, and the abundance in the colon reached 15.97%. However, further analysis at the genus level showed that only one unclassified genus (Lachnospiraceae_UCG-009) in the Lachnospiraceae family with very low relative abundance in the colon showed a significant difference between two groups (Table 4). Therefore, this study could not determine the role of Lachnospiraceae in affecting the UEN of the host.

The synthesized MCP in the colon is not digested and absorbed by the host but excreted with the feces. Therefore, the over-reproduction of colonic microbes might generally result in the reduced UEN of the host. However, surprisingly, this study showed that some colonic archaea had a significant positive correlation with the host UEN, such as Euryarchaeota, Methanomicrobiales and *Methanocorpusculum.* These archaea were all identified as gastrointestinal methanogens (Lopez et al., 1999; Jarvis et al., 2000; Sprenger et al., 2000) and had the ability to promote the decomposition of fiber (Mi et al., 2010). During the course of fiber decomposition, nitrogenous substances bound in fiber could be released and then further decomposed into ammonia or amino acids and absorbed through the intestinal wall (Judson et al., 1975), thus reducing the discharge of fecal nitrogen, which likely explains why these archaea could promote the UEN of the host.

## Conclusion

In summary, obvious individual variation were observed in the UEN of goats. Additionally, the structure of the gastrointestinal microbiota of goats differing in UEN was distinctly different. Rumen microbes had the greatest influence on the host UNE compared with other gastrointestinal sections. Ruminal *Fibrobacter_succinogenes*, *Butyrivibrio_fibrisolvens*, *Ruminococcus*_sp._HUN007, *Prevotella ruminicola* and *Streptococcus bovis* were beneficial for the host utilization of nitrogen.

## Author Contributions

LW, KL, and QP performed the experiments. LW was the principal investigator and contributed to the study design and interpretation of the findings and wrote the manuscript. ZW designed the study. KL analyzed the pyrosequencing NGS reads with LJ and wrote the manuscript. XB wrote the manuscript. All authors read and approved the final version of the manuscript.

## Conflict of Interest Statement

The authors declare that the research was conducted in the absence of any commercial or financial relationships that could be construed as a potential conflict of interest.

## Footnotes

1 http://greengenes.lbl.gov
