# Supplementary figures and images for: Bacterial Community Diversity Associated With Different Utilization Efficiencies of Nitrogen in the Gastrointestinal Tract of Goats
Source: Front Microbiol. 2019 Feb 20;10:239. doi: 10.3389/fmicb.2019.00239 (PMC6401623; doi:10.3389/fmicb.2019.00239)

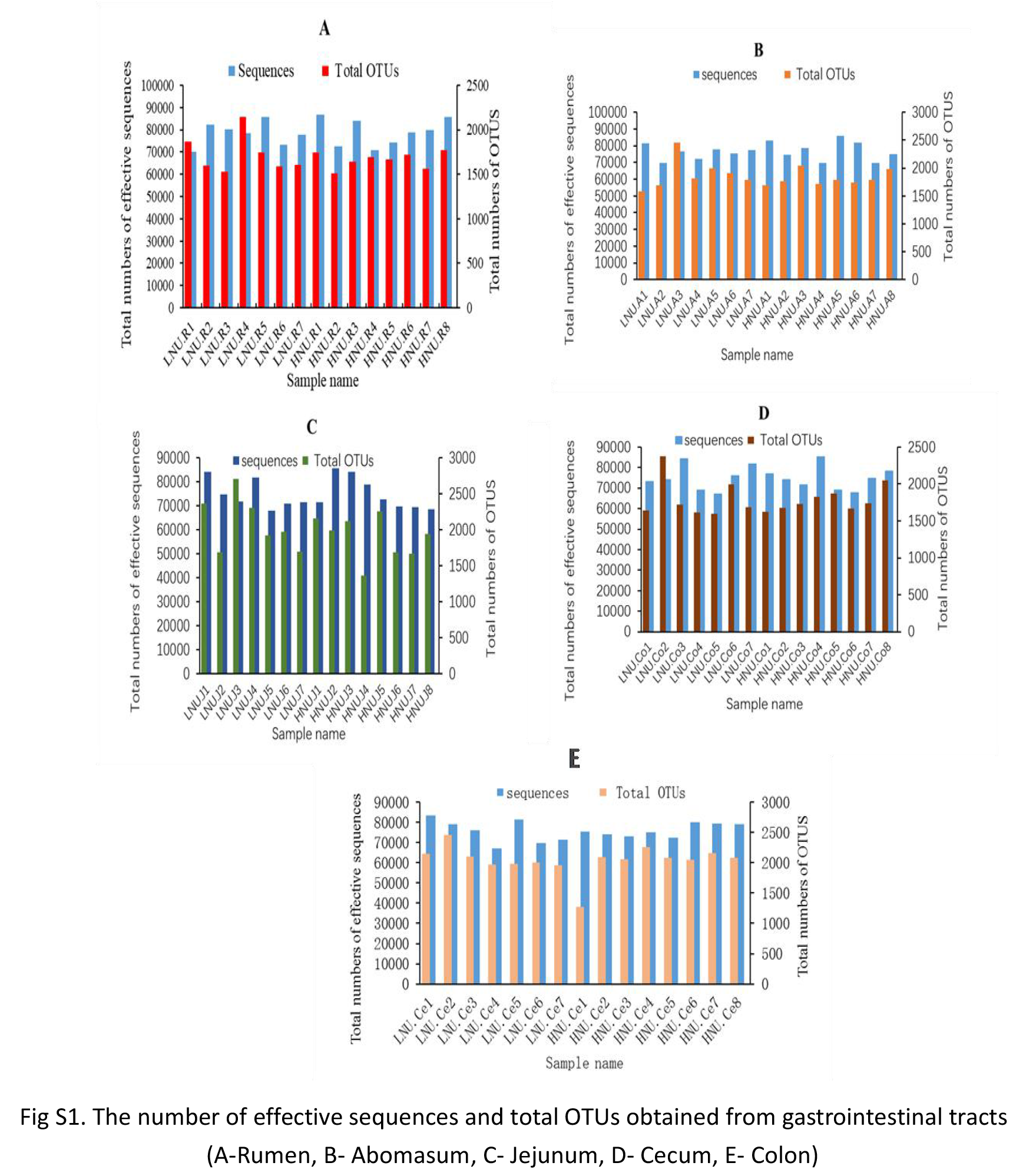

Supplement: Supplementary file 1 [file Image_1.TIF]

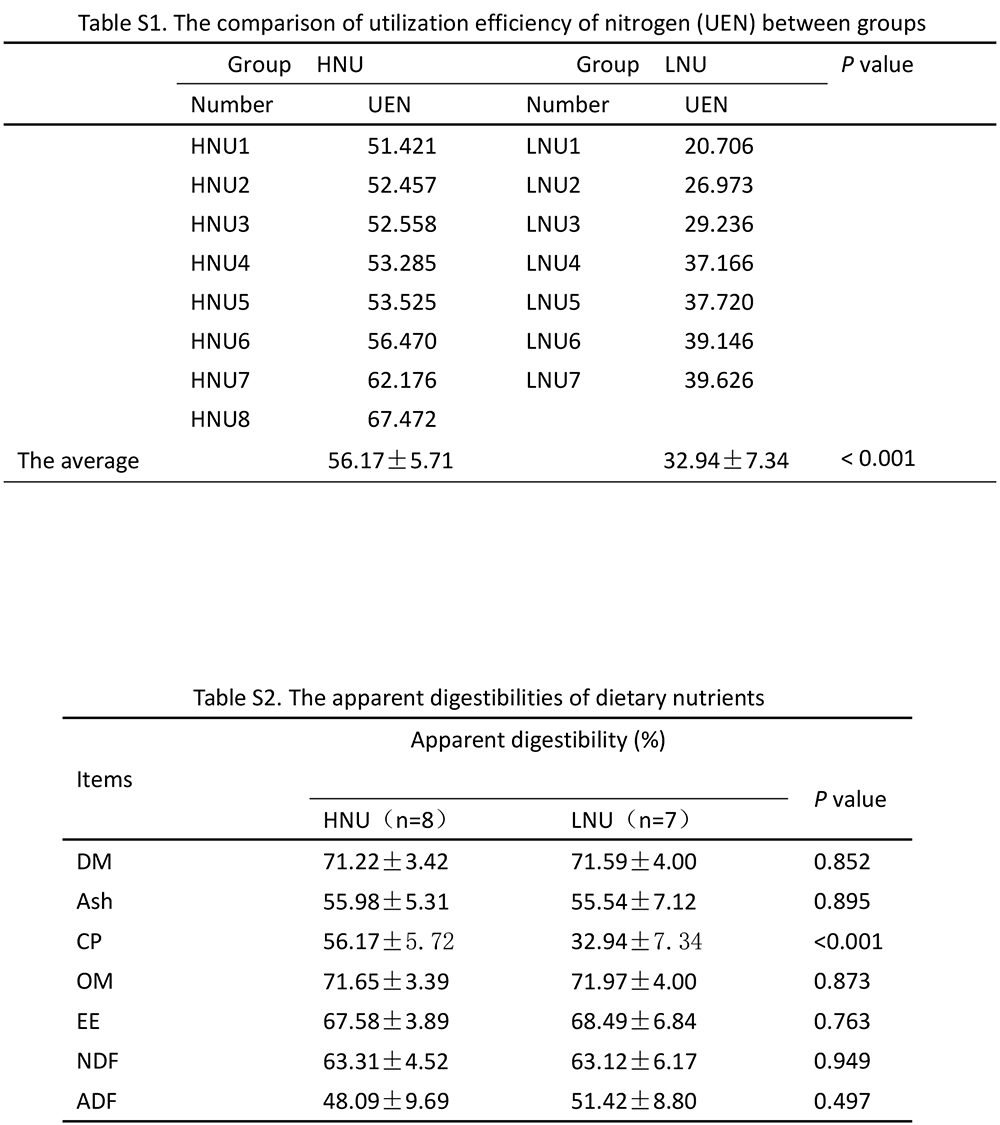

Supplement: Supplementary file 2 [file Image_2.tif]
